# ‘A New Pace of Life’: A Mixed-Methods Exploration of Retirement Plans, Preparations and Experiences in Middle-Aged and Older Autistic and Non-Autistic Adults

**DOI:** 10.1177/13623613261431925

**Published:** 2026-03-29

**Authors:** Zuzanna Kowalczyk, Ahna Huwaida Ahmad Fadzil, Isabel Ward, Francesca Happé, Gavin R Stewart

**Affiliations:** 1 King’s College London, UK

**Keywords:** autism, financial planning, midlife, old age, pension, retirement

## Abstract

Retirement is a major life change affecting routines, finances and wellbeing. Autistic adults may face extra challenges during this transition due to employment barriers, limited support and planning difficulties. However, little is known about their retirement experiences compared to non-autistic adults. This mixed-methods study surveyed 517 adults from the United Kingdom (autistic *n* = 395), aged 40–90 years, about their retirement status, plans, experiences, employment history, income and financial security. Actual or expected retirement ages were similar across groups, but autistic adults were less likely to have made plans and more likely to have plans disrupted by financial, health or personal factors. They also reported lower rates of full-time work, reduced pensions and lower pre-retirement income. Many highlighted a lack of information about pensions and lifestyle planning. Concerns included isolation, loss of routine and financial worries, but some looked forward to more autonomy and time for self-care, leisure activities and interests. While there were many similarities between the autistic and non-autistic groups, our study found that autistic adults may reach retirement differently due to unique work histories, health needs and planning barriers. Tailored, accessible support is needed to help autistic adults plan for financial stability and meaningful post-retirement lives.

**Lay abstract:**

Retirement is a major life change, but very little is known about how autistic adults experience this transition. This study explored retirement experiences/expectations of both autistic and non-autistic adults, finding that retirement happened or was expected to happen at similar ages across groups. However, autistic adults were less likely to plan for retirement and often had more difficulties with jobs, money, pensions and their health. Many felt unsure how to prepare for retirement and wanted clearer information. While some had concerns about isolation and changes to routine, others looked forward to more freedom and time for hobbies. The findings highlight the need for better support to help autistic people plan for retirement.

## Introduction

Retirement refers to the transition period and phase of life when an individual decides to leave regular employment (Ingale & Paluri, 2025). Many retired individuals opt to engage in leisure activities and travel, personal interest pursuits and voluntary or flexible/part-time employment (Genoe et al., 2022). While retirement can occur at any age, it often takes place in later life, coinciding with reaching ‘retirement age’ or having financial means no longer requiring income from employment. In the context of the United Kingdom, retirement age is calculated based on an individual’s year of birth; while this age has gradually increased over time, currently circa 2025, both men and women are eligible to retire with government assistance at age 66 years (Department for Work and Pensions, 2023; Pensions Act, 2007). The U.K. government currently offers a scaling ‘State Pension’ scheme, where an individual can receive up to £221.20 per week (£11,541.90 per annum) if they have contributed to paying 35 or more years of national insurance payments (i.e., a form of income taxation). If an individual does not satisfy this 35-year criterion, their State Pension is scaled accordingly, with a minimum of 10 years’ worth of national insurance payments providing £63.20 per week (£3,297.68 per annum). While some rely fully on their state pension as their only form of income when retired, many people supplement this income with private or workplace pensions or with additional government subsidies and payment schemes.

For many people, retirement is viewed positively as something to look forward to. However, others may not have as favourable an outlook on the prospect of being retired. Given that retirement may involve changes to finances and routines and requires advanced planning, some people, such as those on the autism spectrum, may struggle with the process of retirement (Ingale & Paluri, 2025). We know very little about autism in midlife and older age, with only 0.4% of indexed autism research since 1980 focusing on older adults (Mason et al., 2022). Also, given that many autistic adults are late diagnosed or undiagnosed (O’Nions et al., 2023; Stewart & Happé, 2025), this may cause issues with forward planning for retiring. Furthermore, approximately 30% of autistic adults have been found to have poor ‘normative’ outcomes (Mason et al., 2021) including higher rates of unemployment or under-employment (Buckland, 2024; Davies, Romualdez, et al., 2024), which may result in additional difficulties with the retirement process.

To date, retirement in autistic populations has been a largely neglected topic. To the authors’ knowledge, only two case studies (one published, Hodges et al., 2004; one included in a doctoral thesis, Mason, 2023) and one multi-participant study (Davies, Matthews, et al., 2024) have examined the retirement experiences of autistic people. Davies, Matthews, et al. (2024) conducted a qualitative study interviewing eight women and four men (retired *n* = 4, semi-retired *n* = 4, not-yet-retired *n* = 4) aged 56 to 70 years (mean age = 63 years) about their experiences of planning for and being retired. The researchers produced several themes, which indicated mixed experiences of the retirement process; some participants felt that being autistic (i.e., having good attention to detail, being a logical planner) was beneficial to the retirement planning process, while others found that retirement was something unexpected and involved complex planning and paperwork that was challenging. Others noted that disruptions in their employment history (which affects state pension) had caused issues with retiring, and that they had inadequate support with the planning process. Financial constraints and challenges of balancing care for children and older parents were also raised. Finally, participants highlighted both joy and concerns about the lack of day-to-day structure that being retired would result in, with some expressing worries about losing a sense of self and purpose associated with being employed. Although participants in this study voiced numerous concerns, particularly related to planning and the ‘unexpectedness’ of retirement, only one participant in the study retired at the U.K. retirement age, with most retiring several years prior to this threshold due to ill health or unexpected changes/work-related disputes.

The challenges associated with early/unexpected retirement described by Davies et al. are also found to be experienced in non-autistic populations (e.g., Clarke et al., 2012; de Wind et al., 2013; Schinkel-Ivy et al., 2017), suggesting these experiences are not specific to autistic people and could be associated with early/unexpected retirement rather than retiring at a predetermined age. Given this, there is continued uncertainty about whether autistic people face additional difficulties with the process of retiring. Further research with larger groups of autistic people is necessary to further clarify the extent of retirement-related challenges within this population. In addition, comparative approaches are needed to understand whether any difficulties are unique to autistic people or if they are associated with other challenges experienced regardless of diagnostic group, for example, retiring unexpectedly/below retirement age. By doing so, it will provide further evidence for whether tailored retirement support is needed to ensure autistic people are prepared and supported during this important transition period in later life.

The current study aims to examine the planning and retirement experiences of autistic and non-autistic adults using a mixed-methods approach. Given the dearth of information about retirement in autistic populations, this study is exploratory in nature. A mixed-methods design (combining quantitative group comparisons with qualitative content analysis) was chosen to allow for both the identification of group-level differences and the exploration of participants’ lived experiences in greater depth, providing important context for interpreting quantitative findings in this under-researched area. Comparisons will be made between the autistic and non-autistic groups on a range of topics, including (1) current/pre-retirement employment and financial stability, (2) retirement status, (3) whether plans were made for retirement, (4) financial support and (5) whether there are differences between those who have and have not-yet-retired in the affordability of their living expenses.

## Method

### Study design

This study uses cross-sectional data from the ‘Planning for Old Age’ study, an online survey exploring how autistic and non-autistic people plan for old age and retirement. Prior to the commencement of the study, steering conversations were held via video call with six older adults who have retired (two autistic men, one autistic woman, three non-autistic women) to identify questions that would be relevant for the survey. The research team then created the current study. Steering members were provided with £25 vouchers for their involvement in the steering activities.

The ‘Planning for Older Age’ study was conducted between December 2024 and February 2025. Participants were recruited through study adverts to the ReSpect Lab Participant Database at King’s College London, Autistica’s Research Network and the Cambridge Autism Research Database (CARD). Study adverts referred directly to planning for retirement and being retired. The current study was not advertised on social media due to notable incidents of spam/imposters in autism research (e.g., Lo et al., 2025; Pellicano et al., 2024). Inclusion criteria for the study were being 40 years of age or older (i.e., middle-aged and older; Stewart, 2026), having access to an Internet-enabled device, being able to read English and living in the United Kingdom. The study had no specific exclusion criteria. Participants accessed the survey on Qualtrics. Before starting, participants read an information sheet outlining the study’s aims. Participants gave informed consent and were reminded of their right to withdraw at any time. Participants were then presented with (1) a demographic information questionnaire (including questions about autism and attention-deficit/hyperactivity disorder [ADHD] diagnoses), (2) a series of standardised questionnaires related to traits of autism and ADHD, mental health symptoms, social support and quality of life (not included in this specific publication) and (3) bespoke multiple-choice and open-text questions about their employment history, their retirement status and planning, income and financial stability. All questions were optional but prompted a response if unanswered. Upon completion, participants received a debrief sheet with links to support services. Participants were entered into a prize draw to receive one of ten £25 gift vouchers. All responses were checked for possible spam. Full ethical approval was received for this study through the Health Faculties (Blue) Research Ethics Subcommittee at King’s College London (HR-23/24-45393). The study was not pre-registered.

### Participants

In total, 596 survey responses were recorded, of which 80 (13.4%) were removed due to one or more of not providing consent (*n* = 6, 1.0%); being flagged as duplicate responses by Qualtrics (*n* = 4, 0.7%); having short completion times (less than 10 min; *n* = 26, 4.4%); the survey being incomplete (*n* = 39, 6.5%); not meeting the minimum age requirement (*n* = 4, 0.7%); and living outside of the United Kingdom (*n* = 5, 0.8%). This resulted in a final total of 517 participants (86.6% of the recruited total). Median completion time was 32.5 min.

Participants were aged 40 to 90 years old. Those who disclosed that they had an autism diagnosis (*n* = 359) and those that self-identified as autistic (*n* = 36) were found to have similar endorsement of autistic trait scores and were combined to form the autistic group (total autistic *n* = 395). Participants were asked when they received their autism diagnosis/began to identify as autistic; responses ranged from the current year to 54 years ago as a child (mean years since diagnosis/identity = 10.6 years). People assigned female at birth were diagnosed significantly more recently (*M* = 8.4 years since diagnosis, *SD* = 7.86) than people assigned male at birth (*M* = 13.39 years since diagnosis, *SD* = 7.78), *t*(1,393) = 6.32, *p* < .001. Four (1.1%) participants were diagnosed under 18 years of age. The remaining 122 participants formed a non-autistic comparison group. Groups were broadly matched on age (autistic mean age = 60.35 years; non-autistic mean age = 62.6 years), sex assigned at birth ratio (autistic men = 49.1%; non-autistic men = 47.5%) and living situation. However, some differences were found in other demographic characteristics; the autistic group were more often gender diverse (i.e., non-binary or trans), more likely to endorse a White European ethnicity, more likely to have a postgraduate level of education, more likely to be single or in a non-marital relationship and more likely to live in a council/housing association rented home (i.e., homes that are publicly owned by local government authorities or charities which are offered with long-term affordable rent, often provided based on a financial needs assessment or to support vulnerable and/or people with disabilities). Table 1 shows the demographic characteristics of the autistic and non-autistic groups.

**Table 1.** Demographic characteristics of the autistic and non-autistic groups.

		Autistic group (*n* = 395)		Non-autistic group (*n* = 122)		Group difference	Effect size
Age (years)	*M* (*SD*)	60.35	(11.73)	62.60	(13.44)	*t*(1,515) =-1.78, *p* = .075	*g* =-.38 [-.39, .02]
	[95% CI]	[59.19-61.51]		[60.19-65.01]			
	Min-Max	40-89		40-90			
Sex assigned at birth	Male: female	179: 216		58: 64		χ^2^ = 0.18, *p* = .666	*v* = .02
	%	45.3%: 54.7%		47.5%: 52.5%			
Gender identity	Men: Women: NB/T	175: 194: 26		58: 64: 0		χ^2^ = 8.46, *p* = .015*	*v* = .13
	%	49.1%: 44.3%: 6.6%		47.5%: 52.5%: 0%			
Ethnicity	White	350	(88.6%)^a^	96	(78.7%)^a^	χ^2^ = 19.84, *p* < .001***	*v* = .20
	Black	13	(3.3%)^a^	13	(10.7%)^a^		
	Asian	16	(4.1%)^a^	12	(9.8%)^a^		
	All other ethnicities	16	(4.1%)	1	(0.8%)		
Highest educational qualification	No formal qualifications	5	(1.3%)	0	-	χ^2^ = 50.35, *p* < .001***	*v* = .31
	School to 16	25	(6.3%)^a^	2	(1.6%)^a^		
	School to 18	126	(32.0%)^a^	80	(65.6%)^a^		
	Professional qualifications	27	(6.9%)	4	(3.3%)		
	Undergraduate degree	86	(21.8%)	24	(19.7%)		
	Postgraduate degree	125	(31.7%)^a^	12	(9.8%)^a^		
Marital status	Married / civil partnership	205	(52.0%)	74	(60.7%)	χ^2^ = 11.42, *p* = .022*	*v* = .15
	In a relationship	31	(7.9%)^a^	3	(2.5%)^a^		
	Single	78	(19.8%)^a^	13	(10.7%)^a^		
	Widowed	18	(4.7%)	8	(6.6%)		
	Separated/divorced	62	(15.7%)	24	(19.7%)		
Living situation	Spouse/partner	227	(57.5%)	77	(63.1%)	χ^2^ = 1.22, *p* = .268	*v* *=* .05
	Children	72	(18.2%)	14	(11.5%)	χ^2^ = 3.06, *p* = .080	*v* *=* .08
	Sibling	2	(0.5%)	1	(0.8%)	χ^2^ = 0.16, *p* = .690	*v* *=* .02
	Parent	8	(2.0%)	0	-	χ^2^ = 2.51, *p* = .113	*v* *=* .07
	Other family member	12	(3.0%)	3	(2.5%)	χ^2^ = 0.11, *p* = .739	*v* *=* .02
	Roommate/friend	2	(0.5%)	1	(0.8%)	χ^2^ = 0.16, *p* = .690	*v* *=* .02
	Supported housing	1	(0.3%)	0	-	χ^2^ = 0.31, *p* = .578	*v* *=* .02
	Alone independently	123	(31.1%)	37	(30.3%)	χ^2^ = 0.03, *p* = .865	*v* *<* .01
Homeownership	Own or family-owned home	322	(83.9%)	107	(88.4%)	χ^2^ = 9.09, *p* = .011*	*v* = .13
	Privately renting	30	(7.8%)	13	(10.7%)		
	Renting from council	32	(8.3%)^a^	1	(0.8%)^a^		
Autism diagnosis	Diagnosed	359	(90.9%)	0	-	-	-
	Self-identified	36	(9.1%)	0	-		
Years since autism diagnosis/identity	*M* (*SD*)	10.66	(8.21)	-		-	-
	Min-Max	0-54					

*Note*. NB/T = non-binary and trans; all other ethnicities = Hispanic/Latinx, Middle Eastern/Arab and Mixed/Multiple ethnicities. Effect size calculated using Hedge’s *g* and Cohen’s *v*.

a Cell pairing for response is proportionally different.

* *p* < .05, ** *p* < .01, *** *p* < .001.

### Materials

#### Demographic characteristics

Participants provided detailed demographic information including age, sex assigned at birth, gender, ethnicity, highest educational qualification, marital status, living situation and homeownership (as a proxy for socio-economic status). Participants were also asked if they either (1) had an autism diagnosis (and at what age they received their diagnosis), (2) self-identified as autistic but did not (yet) have an autism diagnosis (and at what age they began to self-identify) or (3) were non-autistic.

#### Employment history and financial stability

Participants were asked to provide an open-text response to describe their employment history, including whether they are currently employed, the types of jobs they have held, whether they were full-time or part-time positions, whether they were supported roles, if they have had any periods of unemployment, and if they have had to stop working due to ill health.

Participants were also asked if they receive(d) a range of employment-related support allowances, which are designed to support people with a disability or condition affecting their daily life or ability to work, either by providing financial assistance, practical workplace support or income replacement. These include Access to Work, Personal Independence Payments, Disability Living Allowance, and Employment and Support Allowance. An open-text option allowed participants to add any other support provisions they may have received.

Participants were also asked a multiple-choice question about whether they feel/felt financially stable prior to retirement (five-point response scale: strongly agree to strongly disagree). A second question asked if they are/were able to put money into savings prior to retirement (response options: no, occasionally, monthly).

#### Retirement and retirement preparations

Participants were asked if they were retired (response options: fully retired, semi-retired, retiring within the next 5 years, retiring in more than 5 years). An open-text option allowed participants to give an alternate response if the other response options did not fit their circumstances. Participants were also asked at what age they retired or at what age they plan to retire.

Participants were also asked if they have (or had) plans for retirement (response options: very detailed plans, somewhat detailed plans, no plans). Based on the response, participants were presented with an open-text box asking either (1) when they began to make plans, if their plans had changed, and if they spoke to family/friends/colleagues/workplace advisors/financial advisors about their retirement plans or (2) if there was a reason why they do not/did not have plans for retirement.

Furthermore, participants were asked if they currently or will receive a range of different types of pensions (e.g., a full/partial U.K. state pension, a workplace pension, a private pension) and different types of pension top-up schemes (e.g., U.K. State Guarantee Credit, U.K. Housing Credits, etc.). An open-text option allowed participants to give an alternative response if the other response options did not fit their circumstances.

#### Current income and affordability of living costs

Participants were asked what their annual income is (response options ranging from ‘£0 to £10,000’, with £5,000 increments up to ‘Over £35,000’ [U.K. median income]). A second question asked if they shared their living expenses with someone else, for example, a partner or family member (response options: yes, no). An open-text option allowed participants to give an alternate response if the other response options did not fit their circumstances.

Participants were also asked a series of questions (response options: all the time, most of the time, some of the time, no) about whether they could afford day-to-day living expenses (e.g., food, drink), monthly living expenses (e.g., mortgage/rent, utility bills), additional monthly expenses (e.g., telephone/Internet bills, TV licence fees) and occasional expenses (e.g., purchasing new clothes, gifts for special occasions).

#### Views on retirement

Participants had the opportunity to respond to three open-text questions about (1) whether there are/were any lifestyle changes they look forward to when retired, (2) any lifestyle changes they are worried about and (3) if there is anything they wish they knew about retirement.

### Data analysis

All statistical analyses were conducted in SPSS (version 25.0; IBM Corp., 2017). Data were cleaned and checked for completeness. Open-text responses to the employment and retirement questions were recoded for consistency. Group differences (autistic vs. non-autistic) in demographics were tested using *t*-tests and chi-square (χ^2^) tests. Employment and retirement group differences were analysed with χ^2^ tests, examining adjusted residuals for proportional differences. To compare income and living cost affordability by group and retirement status, χ^2^ tests were conducted, with adjusted residuals identifying proportional differences between ‘retired’ and ‘not-yet-retired’ subgroups within the autistic and non-autistic groups. The demographic characteristics for the ‘retired’ and ‘not-yet-retired’ were also generated descriptively to contextualise these subsamples (see Supplementary Table 1). Open-text responses about retirement plans, hopes/worries and desired information were analysed using content analysis (a common qualitative method that systematically identifies themes by coding and categorising content into quantifiable data for analysis; Bengtsson, 2016). Multiple comparisons were controlled using the False Discovery Rate (Benjamini & Hochberg, 1995) with an initial α-value of .050. Adjusted α-values were applied by *p*-value rank; all significant results met this threshold.

## Results

### Employment history and financial stability currently/prior to retirement

The autistic group reported significantly lower rates of full-time employment and significantly higher rates of being unemployed or unable to work currently/prior to retirement when compared to the non-autistic group.

The autistic group reported significantly higher rates of receiving Personal Independence Payments, Employment Support Allowances and Universal Credit currently/prior to retirement when compared to the non-autistic group. In addition, the autistic group was significantly less likely to claim no support allowances than the non-autistic group.

The autistic group reported significantly higher rates of low financial stability currently/prior to retirement compared to the non-autistic group. Finally, no statistical differences were found in the ability to save money currently/prior to retirement between the autistic and non-autistic groups (see Table 2).

**Table 2.** Employment History and Financial Status (Currently or Prior to Retirement) of the Autistic and Non-Autistic Groups.

		Autistic group (*n* = 395)		Non-autistic group (*n* = 122)		Group difference	Effect size
Employment (currently/prior to retirement)	In full-time employment	286	(73.9%)^a^	98	(82.4%)^a^	χ^2^ = 13.18, *p* = .010**	*v* *=* *.16*
	In part-time employment	29	(7.5%)	10	(8.4%)		
	Employed, but disruptions due to health	19	(4.9%)	2	(1.7%)		
	Unemployed or unable to work	44	(11.4%)^a^	3	(2.5%)^a^		
	Homemaker	9	(2.3%)	6	(5.0%)		
Financial support allowances (currently/prior to retirement)	Access To work	18	(4.6%)	2	(1.6%)	χ^2^ = 2.13, *p* = .144	*v* *=* .06
	Personal independence payments (PIP)	58	(14.7%)	1	(0.8%)	χ^2^ = 17.72, *p* < .001***	*v* *=* .19
	Disability living allowance	19	(4.8%)	2	(1.6%)	χ^2^ = 2.40, *p* = .121	*v* *=* .07
	Employment support allowance	35	(8.9%)	0	-	χ^2^ = 11.59, *p* *<* .001***	*v* *=* .15
	Universal credit	21	(5.3%)	0	-	χ^2^ = 6.76, *p* = .009**	*v* *=* .11
	None	272	(68.9%)	110^b^	(90.2%)	χ^2^ = 21.83, *p* *<* .001***	*v* *=* .20
Financial stability (currently/prior to retirement)	Low stability	173	(44.1%)	33	(27.0%)	χ^2^ = 11.31, *p* < .001***	*v* = .15
	High stability	219	(55.9%)	89	(73.0%)		
Able to save money (currently/prior to retirement)	No	120	(30.6%)	33	(27.0%)	χ^2^ = 4.61, *p* = .100	*v* = .09
	Occasionally	143	(36.5%)	36	(29.5%)		
	Monthly	129	(32.9%)	53	(43.4%)		

*Note*. Effect size calculated using Cohen’s *v*.

a Cell pairing for response is proportionally different.

b Seven non-autistic participants did not disclose the type of financial support allowances they receive.

* *p* < .05, **p < .01, ***p < .001.

### Retirement status, plans and preparations

In total, 157 autistic participants (41.1%) and 51 non-autistic participants (41.8%) were retired (either fully retired or semi-retired), with the remaining participants being not-yet-retired. Our analyses found that the autistic and non-autistic groups significantly differed in their current retirement status; although they had similar rates of participants who were fully retired or planning to retire, the autistic group reported significantly higher rates of being semi-retired and being of working age but with long-term illness resulting in early medical retirement when compared to the non-autistic group.

No statistical difference was found in actual or planned retirement ages in the autistic and non-autistic groups. In total, 22.3% (*n* = 79) of the autistic group were diagnosed with autism or began to self-identify as autistic prior to their actual/planned retirement age.

The autistic group reported significantly lower rates of having very detailed retirement plans and preparations, as well as significantly higher rates of not having retirement plans and preparations when compared to the non-autistic group.

The autistic group reported significantly lower rates of being eligible to claim a full U.K. state pension and were significantly more likely to be eligible to claim only a partial U.K. state pension when compared to the non-autistic group. In addition, the autistic group reported significantly lower rates of having workplace pensions than the non-autistic group. Comparable rates were found in private pensions, as well as pension top-up schemes in both groups (see Table 3).

**Table 3.** Retirement Status, Age of (Planned) Retirement and Preparations Made by the Autistic and Non-Autistic Groups.

		Autistic group (*n* = 395)		Non-autistic group (*n* = 122)		Group difference	Effect size
Current retirement status	Fully retired	134	(34.2%)	50	(41.0%)	χ^2^ = 14.72, *p* = .012*	*v* *=* *.17*
	Semi-retired	23	(5.9%)^a^	1	(0.8%)^a^		
	Will retire in next 5 years	56	(14.3%)	19	(15.6%)		
	Will retire in more than 5 years	147	(37.5%)	51	(41.8%)		
	Of working age but medically retired	22	(5.6%)^a^	1	(0.8%)^a^		
	Does not intend to retire	10	(2.6%)	0	-		
Retirement age (actual or planned)	Under 60 years old	72	(20.3%)	23	(18.9%)	χ^2^ = 0.18, *p* = .913	*v* *=* .02
	Between 60 to 65 years old	130	(36.6%)	44	(36.1%)		
	Age 66 years or older	153	(43.1%)	55	(45.1%)		
Plans and preparations for retirement	Very detailed plans	85	(21.7%)^a^	51	(41.8%)^a^	χ^2^ = 38.51, *p* *<* .001***	*v* = .27
	Somewhat detailed plans	146	(37.2%)	56	(45.9%)		
	No plans or preparations	161	(41.1%)^a^	15	(12.3%)^a^		
Pension advice sources (among those who made plans)	Friends	139	(60.2%)	97	(90.7%)	χ^2^ = 73.79, *p* *<* .001***	*v* *=* .37
	Family	167	(72.3%)	100	(93.5%)	χ^2^ = 58.79, *p* < .001***	*v* *=* .33
	Colleagues	32	(13.9%)	14	(13.1%)	χ^2^ = 1.31, *p* = .253	*v* *=* .05
	Workplace pension advisors	31	(13.4%)	12	(11.2%)	χ^2^ = 0.48, *p* = .487	*v* *=* .03
	Trade union advisor	17	(7.4%)	10	(9.3%)	χ^2^ = 2.85, *p* = .091	*v* *=* .07
	A financial advisor	137	(59.3%)	82	(76.6%)	χ^2^ = 40.39, *p* < .001***	*v* *=* .28
	Government resources	5	(2.2%)	0	-	χ^2^ = 1.60, *p* = .212	*v* *=* .05
What type of pension do you/will you receive?	Full U.K. state pension	289	(73.2%)	117	(95.9%)	χ^2^ = 28.58, *p* *<* .001***	*v* *=* *.24*
	Partial U.K. state pension	58	(14.7%)	1	(0.8%)	χ^2^ = 17.72, *p* < .001***	*v* *=* .19
	Workplace pension	278	(70.4%)	116	(95.1%)	χ^2^ = 31.37, *p* < .001***	*v* *=* .25
	Private pension	122	(30.9%)	47	(38.5%)	χ^2^ = 2.47, *p* = .116	*v* *=* .07
	Housing credit	28	(7.1%)	9	(7.4%)	χ^2^ = 0.01, *p* = .914	*v* *<* .01
	Warm homes scheme credit	11	(2.8%)	2	(1.6%)	χ^2^ = 0.50, *p* = .480	*v* *=* .03
	Pension credit top-up	8	(2.0%)	1	(0.8%)	χ^2^ = 0.79, *p* = .373	*v* *=* .04

*Note*. Effect size calculated using Cohen’s v.

a Cell pairing for response is proportionally different.

* *p* < .05, ** *p* < .01, *** *p* < .001.

### Current income and affordability of living costs

Among those who are not-yet-retired, the autistic group (*n* = 235, 59% of the autistic sample) reported significantly lower annual income than the non-autistic group (*n* = 71, 53% of the non-autistic sample). No statistical differences were found in annual income in the retired autistic (*n* = 157) and non-autistic (*n* = 51) groups.

The not-yet-retired autistic group were also significantly less likely to share their living expenses with someone else when compared to the not-yet-retired non-autistic group. No statistical difference was found in the rates of sharing living expenses in the retired autistic and non-autistic groups.

For the affordability of living costs, no statistical differences were found between the retired/not-yet-retired autistic and non-autistic groups in day-to-day expenses (e.g., food, drink), monthly living expenses (e.g., rent/mortgage, utility bills) and optional monthly bills (e.g., TV licence, phone and Internet bills). However, the not-yet-retired autistic group and the retired autistic group reported being significantly less able to afford occasional expenses (e.g., new clothes, gifts for special occasions) when compared to their respective non-autistic groups (see Table 4).

**Table 4.** Pension And Financial Profile of the Autistic and Non-Autistic Groups, Split Into Not-Yet-Retired And Retired Subgroups.

		Not-yet-retired				Retired					
		Autistic group (*n* = 235)		Non-autistic group (*n* = 71)		Autistic group (*n* = 157)		Non-autistic group (*n* = 51)		Group difference	Effect size
Annual income	Up to £10,000	40	(17.9%)^a^	4	(5.8%)^a^	21	(14.7%)	8	(16.7%)	Not-yet-retired: χ^2^ = 19.96, *p* = .003** Retired: χ^2^ = 3.70, *p* = .717	*Not-yet-retired: v* = .26 Retired: *v* = .14
	Between £10,001–£15,000	23	(10.3%)^a^	1	(1.4%)^a^	18	(12.6%)	8	(16.7%)		
	Between £15,001–£20,000	21	(9.4%)	3	(4.3%)	16	(11.2%)	5	(10.4%)		
	Between £20,001–£25,000	19	(8.5%)^a^	13	(18.8%)^a^	25	(17.5%)	11	(22.9%)		
	Between £25,001–£30,000	25	(11.2%)	12	(17.4%)	16	(11.2%)	5	(10.4%)		
	Between £30,001–£35,000	27	(12.1%)	12	(17.4%)	12	(8.4%)	5	(10.4%)		
	Over £35,000	68	(30.5%)	24	(34.8%)	35	(24.5%)	6	(12.5%)		
Do you share your living expenses with someone else?	Yes	155	(66.5%)	65	(91.5%)	115	(77.2%)	41	(87.2%)	Not-yet-retired: χ^2^ = 17.04, *p* < .001***	Not-yet-retired: *v* = .24
	No	78	(33.5%)	6	(8.5%)	34	(22.8%)	6	(12.8%)	Retired: χ^2^ = 2.22, *p* = .136	Retired: *v* = .11
Can afford day-to-day expenses (e.g., food, drink)	All the time	96	(41.2%)	41	(57.7%)	92	(59.0%)	30	(58.8%)	Not-yet-retired: χ^2^ = 7.28, *p* = .064 Retired: χ^2^ = 3.78, *p* = .337	Not-yet-retired: *v* = .16 Retired: *v* = .13
	Most of the time	70	(30.0%)	18	(25.4%)	31	(19.9%)	15	(29.4%)		
	Some of the time	54	(23.2%)	11	(15.5%)	27	(17.3%)	5	(9.8%)		
	No	13	(5.6%)	1	(1.4%)	6	(3.8%)	1	(2.0%)		
Can afford monthly living expenses (e.g., rent/mortgage, water/gas/electric bills)	All the time	119	(51.1%)	44	(62.0%)	92	(59.0%)	30	(58.8%)	Not-yet-retired: χ^2^ = 6.58, *p* = .087 Retired: χ^2^ = 7.67, *p* = .053	Not-yet-retired: *v* = .15 Retired: *v* = .19
	Most of the time	63	(27.0%)	21	(29.6%)	31	(19.9%)	14	(27.5%)		
	Some of the time	43	(18.5%)	5	(7.0%)	31	(19.9%)	4	(7.8%)		
	No	8	(3.4%)	1	(1.4%)	2	(1.3%)	3	(5.9%)		
Can afford monthly optional household bills (e.g., TV licence, phone and internet bills)	All the time	117	(50.2%)	39	(54.9%)	88	(56.4%)	28	(54.9%)	Not-yet-retired: χ^2^ = 4.75, *p* = .191 Retired: χ^2^ = 4.45, *p* = .217	Not-yet-retired: *v* = .13 Retired: *v* = .23
	Most of the time	67	(28.8%)	25	(35.2%)	37	(23.7%)	16	(31.4%)		
	Some of the time	38	(16.3%)	5	(7.0%)	27	(17.3%)	4	(7.8%)		
	No	11	(4.7%)	2	(2.8%)	4	(2.6%)	3	(5.9%)		
Can afford occasional expenses (e.g., buying new clothes, gifts for special occasions)	All the time	77	(33.0%)^a^	41	(57.7%)^a^	82	(52.6%)	34	(66.7%)	Not-yet-retired: χ^2^ = 21.21, *p* < .001*** Retired: χ^2^ = 10.51, *p* = .015*	Not-yet-retired: *v* = .26 Retired: *v* = .23
	Most of the time	57	(24.5%)	20	(28.2%)	33	(21.2%)	10	(19.6%)		
	Some of the time	72	(30.9%)^a^	8	(11.3%)^a^	37	(23.7%)^a^	3	(5.9%)^a^		
	No	27	(11.6%)^a^	2	(2.8%)^a^	4	(2.6%)	4	(7.8%)		

*Note*. Effect size calculated using Cohen’s *v*.

a Cell pairing for response is proportionally different.

* *p* < .05, ** *p* < .01, *** *p* < .001.

### Contextualising experiences using content analysis

Participants were asked a series of open-ended questions to contextualise their experiences. Many similarities and shared experiences were found between the autistic and non-autistic groups, although some nuanced differences related to being autistic were raised by autistic participants. The content analysis of the responses provided by the autistic group is presented below, and the content analysis of the non-autistic group is presented in Supplementary Material 1.

### Planning for retirement in the autistic group

Of the 231 autistic participants who made retirement plans, 105 (45%) responded to the open-text question about when they began to plan for retirement, and 123 (53%) responded to the open-text question about whether their plans had substantially changed.

In terms of when retirement plans were made: 55 of the 105 (52%) participants described being ‘early planners’, that is, making plans early in their careers or before the age of 30; 25 of the 105 (23%) participants described being ‘mid-career planners’, that is, beginning to make plans between the ages of 30 and 45 years or when their careers and families were established; 20 of the 105 (19%) participants were ‘later planners’, making plans after the age of 45 years or once retirement was in their nearer future; and 65 of the 105 (62%) participants mentioned that planning for retirement was a gradual process, with their plans developing as retirement approached.

In terms of plans changing: 47 of the 123 (38%) participants noted that their health problems and experiences of burnout had resulted in them changing their plans (e.g., ‘I retired sooner than I anticipated. My health declined significantly even though I strove to lead a very healthy lifestyle, so I had to change my plans.’). In total, 36 of the 123 (29%) participants mentioned that receiving their autism diagnosis had also influenced their plans; several participants reported that their self-understanding had shifted, which resulted in changes to their career and retirement plans (e.g., ‘My autism diagnosis was a surprise. . . it’s made me think about long term career and older age plans.’). In total, 27 of the 123 (22%) participants noted financial issues have changed or delayed their plans, including resulting in needing to work longer to ensure they have pension funds available (e.g., ‘I had to work longer because [we] didn’t save.’). In total, 19 of the 123 (15%) participants mentioned that caring responsibilities for children, parents and partners have changed their plans (e.g., ‘My partner became severely unwell, and I became a carer . . . I burned out which impacted my employment prospects, so I left work.’). In total, 18 of the 123 (15%) participants noted that redundancies or job-related issues linked to COVID have also impacted (positively and negatively) their ability to retire when originally planned (e.g., ‘Redundancy during the pandemic meant I could afford to stop working and retire a few years early.’ and ‘Losing work during COVID caused debt and I am still making up for it now. My retirement will likely look very differently now.’). In total, 17 of the 123 (14%) participants discussed how workplace disputes and bullying resulted in disrupted employment and/or needing to retire early (e.g., ‘I accelerated my retirement plans due to bullying and abuse of power.’). And 14 of the 123 (11%) participants shared that bereavements and relationship breakdowns with their spouses also changed their retirement plans, often through altered financial circumstances or changed housing/living arrangements (e.g., ‘My wife’s death made me rethink how I live and what legacy I leave.’).

Of the 161 autistic participants who did not make retirement plans, 144 autistic participants (89%) responded to an open-text question about why they did not/chose not to make plans. In total, 95 of the 144 (66%) participants noted they have financial constraints or job insecurity, which has impacted their ability to build their pensions (e.g., ‘I couldn’t afford to save into a pension . . .’). In total, 55 of the 144 (38%) participants described that the concept of planning for retirement was overwhelming or that they did not know where to start their planning (e.g., ‘Too much to think about, which is overwhelming.’). Some also highlighted that their executive function issues or mental health conditions have been a barrier to making long-term plans (e.g., ‘I have very poor executive function, and pathological avoidance disorder, and often experience autistic inertia.’). In total, 45 of the 144 (31%) participants reported that they lack an understanding of pensions and/or financial planning (e.g., ‘I have a number of pensions, but I don’t really understand them.’). In total, 30 of the 144 (21%) participants described how they feel it’s too late to begin planning, which was often linked to job instability (e.g., ‘I feel I’m too far behind to catch up.’). However, 20 of the 144 (14%) participants expressed that they hope to never retire (e.g., ‘I now have a part-time job I enjoy and find satisfying, so may not retire at 65 but may want to continue working as long as I can.’).

### Hopes and worries about retirement in the autistic group

When asked about what they are either looking forward to during retirement or enjoy about retirement, 191 autistic participants (48%) responded to the open-text question. In total, 127 of the 191 (67%) participants commented that they look forward to having more free time for relaxation and hobbies and more time for themselves and family (e.g., ‘It will be a new pace of life, which I am greatly looking forward to. Proper time to spend on hobbies and with family.’). In total, 52 of the 191 (26%) participants believed that they would be in better control of their environment when retired (e.g., ‘I won’t have to contend with noisy commutes anymore.’). In total, 46 of the 191 (24%) participants hoped to use their time to benefit their local communities and for volunteering. In total, 20 of the 191 (10%) participants anticipated that retirement would benefit their physical and mental health (e.g., ‘I’ll be able to reallocate all those work hours to more meaningful things, like spending time working on my mental health.’).

When asked if they have any worries for retirement or about retirement, 263 autistic participants (66%) responded to the open-text question. In total, 112 of the 263 (43%) participants reported that they have concerns about their long-term financial security when retired (e.g., ‘I don’t know how I will afford to rent my home and to live the life I live now while working when I am retired.’). In total, 101 of the 263 (38%) participants expressed concerns about losing their sense of purpose, identity and routine (e.g., ‘I worry about the lack of structure, losing my routine, and my purpose in life.’). In total, 95 of the 263 (36%) participants discussed concerns about isolation and loneliness. Some noted how stopping work would reduce their social contact, while others described the feeling of becoming socially invisible (e.g., ‘I worry I will be more isolated than I am now, without work, who will see me.’). In total, 87 of the 263 (33%) participants expressed concerns about ageing more broadly, for example, changes to their health and loss of mobility and cognitive function (e.g., ‘I worry I may lose my sight and my health . . . I may not be able to read, or run, or use my brain.’). In total, 29 of the 263 (11%) participants expressed concerns about needing care, including the prospect of living in a care home (e.g., ‘Being forced into care and support situations that don’t respect my gender or neurodivergence.’). However, 48 of the 263 (18%) participants described that they had no concerns or that they were already happily retired (e.g., ‘No fears. Retiring has been a huge relief . . . there’s always a book to read, a film to watch, or something to do.’).

### Desired information about retirement in the autistic group

When asked if there was any information they wished they knew about planning for retirement and being retired, 212 (54%) autistic participants responded to the open-text question. In total, 88 of the 212 (41%) participants noted that they lack general understanding of what retiring involves and how to prepare for it (e.g., ‘I don’t know what my options are or what I’m supposed to do or who to ask.’). In total, 72 of the 212 (34%) participants described that they are unsure of how pensions work and would like additional information about the different kinds of pensions (e.g., ‘I didn’t know you had to pay into a pension to have income after retirement age.’). In total, 31 of the 212 (17%) participants mentioned they wish they had more autism-specific information about retirement (e.g., ‘The information doesn’t seem relevant for me as an autistic person, like what support will be available’.). However, 28 of the 212 (13%) participants reported they feel satisfied with their understanding of retirement.

## Discussion

This mixed-methods study documents the experiences of planning for and being retired among a large group of autistic and non-autistic adults in midlife and older age living in the United Kingdom. Similarities were found between groups, such as comparable retirement ages that mirror the U.K. state pension age. However, there were also significant differences in retirement preparation, employment history, pension eligibility and income. Open-text responses contextualised this further, highlighting the impact of financial difficulties, health challenges and disrupted employment that autistic participants experienced when planning for and transitioning into retirement with stability and confidence.

When considering timing and preparations for retirement, findings from our large-scale quantitative study found no significant difference between autistic and non-autistic participants in actual or expected retirement ages. This is contrary to the qualitative findings of Davies, Matthews, et al. (2024). When considering why this difference could be, the sampling approach could be an important factor. Our study included almost 400 autistic people who were recruited via established research participant databases, while Davies et al. was a smaller-scale qualitative study involving 12 autistic people who were recruited through social media. While both our study and Davies et al. mentioned retirement in the study adverts, our study had a broader focus about planning for older age; therefore, we may have a broader representation of experiences in our sample. However, our study did have a minority of autistic participants who did retire early for similar reasons highlighted in Davies et al. (e.g., health and workplace-related difficulties), suggesting that for some autistic people, early and/or unplanned retirement may be a necessity.

Another finding of our study was that the pathway into retirement for autistic people may differ from their non-autistic peers, with our autistic participants having more varied levels of planning and preparedness for retirement than the non-autistic participants. Open-text responses indicated that many autistic participants experienced frequent changes to their retirement plans, most often due to health issues, financial strain or shifting personal circumstances. The influence of a late autism diagnosis was also evident, with some participants describing it as a turning point that reframed their understanding of their own needs and future plans. These sentiments mirror findings from Davies et al., whose participants also reported changes to plans and the impact of a later-life diagnosis.

Moreover, autistic participants were significantly more likely to report having made no retirement plans at all. Open-text responses described feelings of being overwhelmed by the process, unsure where to start or excluded from the possibility of retirement due to financial barriers, which are also sentiments shared in Davies, Matthews, et al. (2024). Hershey et al. (2012) propose that effective retirement planning depends on three key components: capacity, opportunity and willingness. When these components are compromised, planning may be impeded. While originally focused on finances, this framework may help explain barriers faced by autistic individuals in broader retirement planning, as challenges reported in this study and wider research may negatively impact its key components. For example, opportunity and capacity may be limited by financial insecurity linked to under-employment and employment challenges (Buckland, 2024; Davies, Romualdez, et al., 2024) or higher rates of health problems and unmet health needs (Mansour et al., 2025; O’Nions et al., 2024). Executive functioning differences (Demetriou et al., 2018; Olde Dubbelink & Geurts, 2017; Stewart et al., 2018, 2023) and social difficulties that limit support networks (Black et al., 2024; Charlton et al., 2023; Francis et al., 2025) and increased experiences of loneliness and social isolation (Grace et al., 2022; Stewart et al., 2024) may also make planning more daunting. While willingness is one of the three components in the model, it is important to note that planning avoidance should not be mistaken for a lack of motivation. Instead, it may reflect a rational response to the external barriers, uncertainty or previous experiences of exclusion from financial and employment systems. This interpretation is supported in our study by qualitative accounts in which participants described feeling overwhelmed or disconnected from the concept of retirement, rather than disinterested in preparing for it.

When examining the financial aspects of retirement (i.e., employment history, pension access and income disparity), we found both similarities and differences between groups. Autistic participants reported significantly lower rates of full-time employment and higher rates of unemployment or being unable to work than non-autistic participants, reflecting broader employment challenges faced by autistic people (as reported in Buckland, 2024). These employment barriers have clear long-term impacts on financial security. Autistic participants were less likely to qualify for the full state pension and more likely to receive a reduced rate. They also reported lower workplace pension participation than non-autistic participants and national averages (Department for Work and Pensions, 2024).

These patterns highlight systemic issues: autistic workers tend to earn less, are more likely to be underemployed and face persistent income gaps even with higher education qualifications (Vincent & Ralston, 2024). Barriers include inaccessible recruitment, inflexible workplaces, sensory challenges and discrimination (Buckland, 2024; Davies, Romualdez, et al., 2024). Many autistic workers are in non-permanent roles without pension benefits, and fear of stigma can discourage disclosing a diagnosis, limiting access to workplace adjustments and stable career paths.

Income disparities were also reflected in our findings. Among non-retired participants, the autistic group reported significantly lower annual incomes and were less likely to share living expenses with others (despite having similar rates of marital status and co-habitation to the non-autistic group). Although both groups reported similar abilities to afford essential costs such as rent and food, autistic participants, regardless of retirement status, were significantly less likely to afford occasional or non-essential expenses such as gifts or new clothing. While individual circumstances such as location, expenses or having dependants may affect living affordability, this finding could indicate reduced financial flexibility among autistic participants. In addition, it may also suggest that married/cohabiting autistic people may be more likely to keep their finances separate from their partners, perhaps due to this income being linked to government-based financial support allowances (e.g., Personal Independence Payments).

The final findings extend beyond the logistical and financial aspects of retirement, highlighting hopes, concerns and the need for more holistic, tailored resources. Many autistic participants looked forward to retirement as a chance for greater autonomy, comfort and time for hobbies or volunteering, and as relief from stressful work environments, which were also views often shared by non-autistic participants (presented in Supplementary Material 1). Some autism-specific points were also raised by autistic participants, such as having greater control over their environments to reduce their sensory overwhelm (Chen et al., 2024). However, many autistic participants also worried about losing daily structure and a sense of identity without work, and about increased loneliness, reflecting broader evidence that autistic people experience higher loneliness across the lifespan (Francis et al., 2025; Grace et al., 2022; Stewart et al., 2024). Financial insecurity was another common concern, supported by differences in employment and pension accrual found in this study. Furthermore, a key barrier to effective planning was the lack of accessible, relevant information. While some felt well-informed, many reported gaps in financial literacy and general retirement knowledge. These findings have important implications for ageing and retirement policies for autistic adults.

To support positive retirement experiences, tailored, accessible and affirmative resources are needed to address the specific retirement hopes and concerns of autistic people. Currently, U.K. retirement resources mostly focus on financial guidance (e.g., AgeUK, n.d.; UK Government, n.d.), which, while important, is insufficient on its own (a sentiment found in our study, as well as Davies, Matthews, et al., 2024). As such, a more holistic approach is needed, covering issues such as social connection, maintaining routines, and building identity and purpose beyond employment.

When interpreting this study’s findings, it is important to consider its strengths and limitations. A key strength is the involvement of middle-aged and older autistic individuals in steering interviews during the design phase. Although full co-production would have been ideal, it was not feasible due to limited funding. Another strength is the large participant group, recruited through established autism research networks, reducing the risk of spam or imposters. We also limited participation to U.K. residents to ensure consistency in retirement context. In addition, our mixed-methods approach combined quantitative survey data and open-text questions, enabling group comparisons while allowing participants to share their experiences in their own words.

However, there are limitations. Conducting the study online may have excluded people not on the Internet, as well as those with intellectual disabilities or higher support needs, limiting the diversity of perspectives. The sample was also predominantly White, well-educated and diagnosed/self-identified with autism later in life. While the U.K.-focus increased consistency, it limits generalisability to other countries. The study’s cross-sectional design means participants were at different stages of retirement: younger participants saw retirement as distant, while older participants may have recalled prior plans inaccurately. Both groups reported higher rates of full state pension eligibility than national averages (Department for Work and Pensions, 2025), suggesting that participants may have been more interested or engaged in retirement planning than the general population. Also, while our questions were comprehensive, as this study was conducted as a survey, we could not prompt for additional information about how their experiences may connect (e.g., to gain further insight into their employment history and their workplace pensions, how certain life experiences (e.g., health problems, marital breakdown, bereavement) may have disrupted their career and retirement, etc.). In addition, we opted not to examine sex differences in this study, as we were mindful of statistical power and the already lengthy number of analyses required for this publication. Finally, as an estimated 90% of autistic adults over 40 are undiagnosed (O’Nions et al., 2023; Stewart & Happé, 2025), the experiences of undiagnosed individuals (who may have less access to support) are likely under-represented in this study.

Despite these limitations, this is the first large-scale study of retirement experiences in an autistic population using both quantitative and qualitative data. We recommend future research use longitudinal methods to examine how retirement planning and experiences develop over time for autistic adults, including those with high autistic traits who may be undiagnosed. This would help track changing plans in response to life circumstances and provide deeper insight into how factors like financial stability, housing, health and location shape retirement over time. Given the complexity and variability of retirement, longitudinal designs are well-suited to capture this nuance. In addition, future studies should consider whether experiences are specific to autistic populations (or neurodivergent populations more broadly), as well as account for potential confounding factors such as poor mental health and the intersecting experiences of other marginalised groups, including people with chronic health conditions, people with disabilities and individuals facing broader social, economic or health-related disadvantage.

In conclusion, this large-scale mixed-methods study explored experiences of planning for and being retired in a large group of autistic and non-autistic adults in midlife and older age living in the United Kingdom. While retirement age did not significantly differ between groups, the pathways into retirement were notably different. Autistic participants reported lower rates of full-time employment, reduced pension access and lower income prior to retirement. They were also less likely to have made retirement plans, and among those who had plans, they were more likely to have been disrupted or changed. These findings highlight the need for more inclusive and accessible retirement resources that account for the realities of neurodiverse lives. With the right support, retirement can offer autistic adults’ greater autonomy and opportunities for meaningful, fulfilling activity. However, this potential can only be realised if planning resources and employment structures are designed to be responsive and attuned to autistic people’s needs.

## Supplemental Material

sj-docx-1-aut-10.1177_13623613261431925 – Supplemental material for ‘A New Pace of Life’: A Mixed-Methods Exploration of Retirement Plans, Preparations and Experiences in Middle-Aged and Older Autistic and Non-Autistic Adults Supplemental material, sj-docx-1-aut-10.1177_13623613261431925 for ‘A New Pace of Life’: A Mixed-Methods Exploration of Retirement Plans, Preparations and Experiences in Middle-Aged and Older Autistic and Non-Autistic Adults by Zuzanna Kowalczyk, Ahna Huwaida Ahmad Fadzil, Isabel Ward, Francesca Happé and Gavin R Stewart in Autism
