# Supplementary material for: ‘A New Pace of Life’: A Mixed-Methods Exploration of Retirement Plans, Preparations and Experiences in Middle-Aged and Older Autistic and Non-Autistic Adults
Source: Autism. 2026 Mar 29;30(5):1330–43. doi: 10.1177/13623613261431925 (PMC13087168; doi:10.1177/13623613261431925)
Supplement: sj-docx-1-aut-10.1177_13623613261431925 – Supplemental material for ‘A New Pace of Life’: A Mixed-Methods Exploration of Retirement Plans, Preparations and Experiences in Middle-Aged and Older Autistic and Non-Autistic Adults [file sj-docx-1-aut-10.1177_13623613261431925.docx]

**“*A new pace of life*”: A mixed-methods exploration of retirement plans, preparations and experiences in middle-aged and older autistic and non-autistic adults.**

| *Supplementary Table 1. Demographic characteristics of the autistic and non-autistic groups, split by retirement status.* | | | | | | | | | |
| --- | --- | --- | --- | --- | --- | --- | --- | --- | --- |
|  |  | **Not-Yet-Retired** | | | | **Retired** | | | |
|  |  | **Autistic group (n=235)** | | **non-Autistic group (n=71)** | | **Autistic group (n=157)** | | **non-Autistic group (n=51)** | |
| **Age (years)** | *M (SD)* | 53.31 | (7.98) | 53.25 | (8.25) | 71.06 | (7.74) | 75.61 | (6.72) |
|  | *[95% CI]* | [52.28-54.34] | | [51.30-55.21] | | [69.84-72.28] | | [73.72-77.50] | |
|  | *Min-Max* | 40-78 | | 40-70 | | 49-89 | | 63-89 | |
| **Sex Assigned at Birth** | *Male : Female* | 79 : 156 | | 32 : 39 | | 100 : 57 | | 26 : 25 | |
|  | *%* | 33.6% : 66.4% | | 45.1% : 54.9% | | 63.7% : 36.3% | | 51.0% : 49.0% | |
| **Gender Identity** | *Men : Women : NB/T* | 77 : 138 : 20 | | 32 : 39 | | 98 : 53 : 6 | | 26 : 25 | |
|  | *%* | 32.8% : 58.7% : 8.5% | | 45.1% : 54.9% | | 62.4% : 33.8% : 3.8% | | 51.0% : 49.0% | |
| **Ethnicity** | *White* | 195 (83.0%) | | 48 (67.6%) | | 152 (96.8%) | | 48 (94.1%) | |
|  | *Black* | 11 (4.7%) | | 11 (15.5%) | | 2 (1.3%) | | 2 (3.9%) | |
|  | *Asian* | 16 (6.8%) | | 11 (15.5%) | | 0 - | | 1 (2.0%) | |
|  | *All other ethnicities* | 13 (5.5%) | | 1 (1.4%) | | 3 (1.9%) | | 0 - | |
| **Highest educational qualification** | *No formal qualifications* | 4 (1.7%) | | 0 - | | 1 (0.6%) | | 0 - | |
|  | *School to 16* | 16 (6.8%) | | 1 (1.4%) | | 9 (5.7%) | | 1 (2.0%) | |
|  | *School to 18* | 63 (26.8%) | | 39 (54.9%) | | 63 (40.1%) | | 41 (80.4%) | |
|  | *Professional qualifications* | 17 (7.2%) | | 3 (4.2%) | | 9 (5.7%) | | 1 (2.0%) | |
|  | *Undergraduate degree* | 53 (22.6%) | | 20 (28.2%) | | 33 (21.0%) | | 4 (7.8%) | |
|  | *Postgraduate degree* | 81 (34.5%) | | 8 (11.3%) | | 42 (26.8%) | | 4 (7.8%) | |
| **Marital status** | *Married / civil partnership* | 116 (49.4%) | | 45 (63.4%) | | 87 (55.4%) | | 29 (56.9%) | |
|  | *In a relationship* | 21 (8.9%) | | 2 (2.8%) | | 10 (6.4%) | | 1 (2.0%) | |
|  | *Single* | 48 (20.4%) | | 2 (2.8%) | | 29 (18.5%) | | 11 (21.6%) | |
|  | *Widowed* | 9 (3.8%) | | 6 (8.5%) | | 9 (5.7%) | | 2 (3.9%) | |
|  | *Separated / divorced* | 40 (17.0%) | | 16 (22.5%) | | 22 (14.0%) | | 8 (15.7%) | |
| **Living situation** | *Spouse / partner* | 125 (53.2%) | | 46 (64.8%) | | 100 (63.7%) | | 31 (60.8%) | |
|  | *Children* | 64 (27.2%) | | 13 (18.3%) | | 7 (4.5%) | | 1 (2.0%) | |
|  | *Sibling* | 0 (0.0%) | | 0 (0.0%) | | 2 (1.3%) | | 1 (2.0%) | |
|  | *Parent* | 7 (3.0%) | | 0 (0.0%) | | 1 (0.6%) | | 0 (0.0%) | |
|  | *Another family member* | 4 (1.7%) | | 2 (2.8%) | | 8 (5.1%) | | 1 (2.0%) | |
| Note: NB/T = Non-binary and trans; All other ethnicities = Hispanic/Latinx, Middle Eastern/Arab, and Mixed/Multiple ethnicities. | | | | | | | | | |

**Supplementary Material 1.**

Content analysis of non-autistic participant responses to open-text questions.

***Planning for Retirement in the non-autistic group (content analysis)***

Of the 107 non-autistic participants who made retirement plans, 67 (62%) responded to the open-text question about when they began to plan for retirement, and 58 (54%) responded to the open-text question about whether their plans had substantially changed.

In terms of when retirement plans were made: 28/67 (42%) described being ‘early planners’, i.e., making plans early in their careers or before the age of 30. 15/67 (23%) described being ‘mid-career planners’, i.e., beginning to make plans between the age of 30-45 or when their careers and families were established. 18/67 (26%) were ‘later planners’, making plans after the age of 45 or once retirement was in their nearer future. 40/67 (60%) participants mentioned that planning for retirement was a gradual process, with their plans developing as retirement approached. Additionally, 15/67 (23%) mentioned that their partner/spouse or family were also involved in the planning process, either influencing decisions or taking lead on the planning.

In terms of plans changing: 16/58 (28%) noted that their family circumstances have influenced their retirement plans (e.g., “*There have been some issues with family health, so that has caused set-backs.*”). 13/58 (23%) mentioned that COVID had either accelerated or disrupted their retirement plans (e.g., “*I retired a bit earlier than planned due a mix of COVID and discovering I could.*” and “*Being made redundant during COVID has maybe set me back a few years.*”). 4/58 (7%) mentioned they retired early due to health issues (e.g., “*Health problems caused me to go into early semi-retirement.*”). 15/58 (26%) noted that their plans had not changed and they will or have been able to retire without making adjustments to their existing plans.

Of the 15 non-autistic participants who did not make retirement plans, 12 participants (80%) responded to an open-text question about why they did not / chose not to make plans. 8/12 (67%) mentioned that retirement felt too far in the future to plan for (e.g., “*It’s a ‘counting chickens before they hatch’ situation. It's so far off, hard to make plans on such a big scale. I'll retire when I retire...*”). 5/12 (42%) noted that they did not feel like they need to make plans for retirement. 2/12 (17%) noted that they will begin making plans in the future (e.g., “*I’ll probably start seriously making plans in a few years.*”).

***Hopes and Worries about Retirement in the non-autistic group (content analysis)***

When asked about what they are either looking forward to during retirement or enjoy about retirement, 48 non-autistic participants (40%) responded to the open-text question. 16/48 (34%) noted that they look forward to the freedom that retirement will being (e.g., “*Not having to work anymore is the dream.*”). 14/48 (30%) mentioned that they see retirement as a time where they can pursue leisure pursuits, for example hobbies, nature, arts, gardening, or simply having more personal time (e.g., “*My retirement has been centred around family and fun, cooking and baking, rambling and nature, spending time with pets and loved ones.*”). 10/48 (21%) noted they desire to travel more when they are no longer working (e.g., “*I’ll be working less and going away on holiday more.*”). 8/48 (17%) shared that they hope to spend time with younger family members (e.g., “*I will be spending time with my children, and maybe some grandchildren too.*”). 5/48 (11%) noted they hope retirement will be beneficial to their health and stress-levels (e.g. “*I'd like to feel less stressed and exhausted.*”). 3/48 (7%) acknowledged that their hopes for retirement had not been fulfilled (e.g., “*We had hoped for freedom and adventure, alas it has not happened.*”).

When asked if they either have any worries for retirement or about retirement, 62 non-autistic participants (51%) responded to the open-text question.

28/62 (46%) shared that they fear losing their sense of purpose (through loss of work and also changing family roles), resulting in them becoming lonely or isolated (e.g. “*I’m worried about feeling lost and without a purpose*” and “*I’m worried that the kids will move out and I'll be left in a big house with just my wife.*”). 18/62 (29%) discussed having concerns about their health changing, and generally worries about ageing (e.g. “*That retirement will just be ill health, disability, death, bereavement, and dementia.*”). 5/62 (8%) mentioned concerns about their long-term financial security with only a pension (e.g., “*My resources are finite and I’ll need to be mindful of that.*”). However, 25/62 (40%) mentioned that they have no fears or concerns.

***Desired Information about Retirement in the non-autistic group (content analysis)***

When asked if there was any information they wished knew about planning for retirement and being retired, 52 non-autistic participants (43%) responded to the open-text question.

23/52 (45%) shared they wanted to have more thorough information about different types of pensions and what their collective pensions (i.e., state and personal pensions) would equate to in monthly income (e.g. “*I have no idea how to work out what my monthly income will actually be*.”). 12/52 (23%) have found that most information doesn’t take into account when things ‘go wrong’, resulting in further worries about retirement (e.g. “*Guides are focus on ‘blue sky’ retirement plans [i.e., the ideal situation], but there is very little information about what to do when things don’t go to plan.”*). 3/52 (6%) noted that most pension information is tailored towards couples, rather than single people (e.g. “*It’s not a system that is kind to single people, and most information overlooks us.*”). However, 27/52 (52%) noted they feel satisfied with their understanding of retirement
